# External validation of clinical prediction rules for complications and mortality following *Clostridioides difficile* infection

**DOI:** 10.1371/journal.pone.0226672

**Published:** 2019-12-17

**Authors:** Catherine Beauregard-Paultre, Claire Nour Abou Chakra, Allison McGeer, Annie-Claude Labbé, Andrew E. Simor, Wayne Gold, Matthew P. Muller, Jeff Powis, Kevin Katz, Suzanne M. Cadarette, Jacques Pépin, Louis Valiquette

**Affiliations:** 1 Department of Microbiology and Infectious Disease, Université de Sherbrooke, Sherbrooke, Québec, Canada; 2 Mount Sinai Hospital, Toronto, Ontario, Canada; 3 Division of Infectious Disease and Microbiology, CIUSSS de l’Est-de-l’Ile-de-Montréal, Montréal, Québec, Canada; 4 Microbiology, Sunnybrook Health Sciences Centre, Toronto, Ontario, Canada; 5 Toronto General Hospital, Toronto, Ontario, Canada; 6 St.Michael’s Hospital, Toronto, Ontario, Canada; 7 Michael Garron Hospital, Toronto, Ontario, Canada; 8 Department of Infection Control, North York General Hospital, Toronto, Ontario, Canada; 9 Leslie Dan Faculty of Pharmacy and Dalla Lana School of Public Health, University of Toronto, Toronto, Ontario, Canada; 10 Centre de Recherche du Centre Hospitalier Universitaire de Sherbrooke, Sherbrooke, Québec, Canada; University of Maryland School of Medicine, UNITED STATES

## Abstract

**Background:**

Several clinical prediction rules (CPRs) for complications and mortality of *Clostridioides difficile* infection (CDI) have been developed but only a few have gone through external validation, and none is widely recommended in clinical practice.

**Methods:**

CPRs were identified through a systematic review. We included studies that predicted severe or complicated CDI (cCDI) and mortality, reported at least an internal validation step, and for which data were available with minimal modifications. Data from a multicenter prospective cohort of 1380 adults with confirmed CDI were used for external validation. In this cohort, cCDI occurred in 8% of the patients and 30-day all-cause mortality occurred in 12%. The performance of each tool was assessed using individual outcomes, with the same cut-offs and standard parameters.

**Results:**

Seven CPRs were assessed. Three predictive scores for cCDI showed low sensitivity (25–61%) and positive predictive value (PPV; 9–31%), but moderate specificity (54–90%) and negative predictive value (NPV; 82–95%). One model [using age, white blood cell count (WBC), narcotic use, antacids use, and creatinine ratio > 1.5× the normal level as covariates] showed a probability of 25% of cCDI at the optimal cut-off point with 36% sensitivity and 84% specificity. Two scores for mortality had low sensitivity (4–55%) and PPV (25–31%), and moderate specificity (71–78%) and NPV (87–92%). One predictive model for 30-day all-cause mortality [Charlson comorbidity index, WBC, blood urea nitrogen (BUN), diagnosis in ICU, and delirium] showed an AUC-ROC of 0.74. All other CPRs showed lower AUC values (0.63–0.69). Errors in calibration ranged from 12%- 27%.

**Conclusions:**

Included CPRs showed moderate performance for clinical use in a large validation cohort with a majority of patients infected with ribotype 027 strains and a low rate of cCDI and mortality. These data show that better CPRs need to be developed and validated.

## Introduction

*Clostridioides difficile* infection (CDI) is a significant nosocomial infection, accounting for 10–25% of antibiotic-associated diarrhea [1]. Approximately, 20% of patients with CDI will experience a complicated disease course, which is defined by the presence of hypotension or shock, ileus, or megacolon [2]. Mortality is twice as high in hospitalized patients with CDI than in outpatients [3]. The emergence of the hypervirulent strain NAP1/BI/027 in the early 2000s has been associated with an increase in unfavorable outcomes [4–6]. In many cases, severe and complicated CDI (cCDI) leads to extended hospital stays and surgical procedures [3], further exacerbating the burden of this disease.

Many studies have tried to identify risk factors for cCDI including age, leukocytosis, high C-reactive protein levels, hypoalbuminemia, acute renal failure and comorbidities such as diabetes, chronic kidney injury, and immunosuppression [2]. Many clinical prediction rules (CPRs) for severe CDI and mortality have been developed, but few have been validated in multiple clinical settings where prevalence and outcomes vary. Consequently, no CPRs have been widely accepted for clinical use. To build a clinically relevant prediction rules, many essential steps are needed. The first step is derivation, which consists of identifying variables with predictive power, usually by multivariable analysis in the original cohort. The second step is internal validation, which is performed on a subset of the original cohort (or with resampling techniques such as the bootstrapping method). This step determines the reproducibility of the rules by demonstrating the stability of the selection of the predictors and quality of predictions. However, when internal validation is performed on the same cohort used for derivation, it usually overestimates the performance of scores [7]. Consequently, the third step is a broad validation (external validation) to evaluate the performance of the rules on a different cohort from a separate clinical setting, with different prevalence and disease outcomes. The last step is an impact analysis to evaluate how the rule is used in real-life, and how it impacts clinician behaviour and clinical outcomes is the last essential step for translation from research to clinic [8]. An accurate predictive tool would be useful for early recognition of patients who are at higher risk of unfavorable outcomes, and for improved stratification of patients in clinical trials. In this context, we conducted an external validation of selected CPRs for cCDI and CDI mortality.

## Methods

### Systematic review

Published CPRs were identified through a systematic review. An initial review [9] was conducted on studies from 1978 to 2011, according to PRISMA guidelines and using an electronic research of databases (MEDLINE, PubMed, Embase, Web of science and Cochrane library for evidence-based medicine) and conference proceedings [10]. An updated review was performed for studies through December 2018, using MEDLINE and PubMed databases, using the same steps and the same combination of keywords as the initial review. The keywords were: “(Clostridium difficile or Clostridium difficile-associated diarrhea or Clostridium difficile-associated disease) AND (diarrhea or diarrhoea or colitis or pseudomembranous or enterocolitis or enteritis or antibiotic-associated disease) AND (sensitivity or specificity or prediction or index or score or model or factor or gradient or decision rule or decision technique or prognosis or risk index or risk score or risk model or risk scale).”

We included publications on CPRs, scoring systems (point systems generated by comparing the parameter estimates of a multivariable regression model), or predictive multivariable models for cCDI and CDI mortality in general patients. Study inclusion criteria were as follows: i) a clear methodology for derivation, ii) an internal validation step, and iii) having predictors available integrally or with minimal adaptation in the validation cohort. Publications studying specific populations (e.g. patients with immunosuppression, inflammatory bowel disease, or children) were excluded.

### External validation of included CPRs

Data on 1380 adults with confirmed CDI from a multicenter prospective cohort with a 90-day follow-up period were used for the external validation of selected CPRs. Patients were enrolled in 10 Canadian hospitals across two provinces (2005–2008). Detailed methods are as previously described [11]. Descriptive data on the cohort are provided in the Supplementary materials (S1 Table). Data from the validation cohort were recoded to reproduce predictors and primary outcomes of each included CPR, and modifications were made to match available data (Table 1). Our data were independent from the data used in the derivation of any of the included CPRs. Patients’ characteristics in sub-cohorts used for each CPR are also described in S1 Table. Patients with missing data for any predictor were excluded from analyses. The missingness of predictors was most likely a random event and was not associated with the outcome. The same point assignment and cut-off values were used for validation analyses.

**Table 1:** Characteristics and methodology of included studies, and modifications in the external validation cohort.

Study, description of derivation and reported validation methods	Outcomes	Criteria in score or model	Reported aOR (95%CI)	Assigned points^a^	Range (points)	Modifications in the external validation cohort
**Prediction of cCDI**						
**Na et al. 2015 [25]**Prospective cohort of hospitalized patients, one center for derivation (2004–2006), USA. - Follow-up: not reported, events occurring during same hospital stay as enrollment CDI. - Multivariable logistic regression. - Validation cohorts: inpatients, one center in Dublin (2007–09) and one in Houston, Texas (2006–10).	**Severe CDI** - ICU admission attributable to CDI or CDI a contributing factor^b^ - Toxic megacolon - Colectomy attributable to CDI - Death attributable to CDI or CDI as a contributing factor^b^	Age ≥ 65 years	2.39 (1.06–5.39)	1	0 to 3	Colectomy or hemicolectomy for any indication
		WBC ≥ 20 x 10^3^ cells/μL	4.21 (2.06–8.62)	1		
		Creatinine ≥ 2 mg/dL	8.12 (2.51–26.27)	1		
			
**Hensgens et al. 2014 [26]** Multicentric (n = 9) prospective cohort: inpatients (2006–09), The Netherlands - Follow-up: 30 days. - Multivariable logistic regression. - Validation: bootstrapping (n = 200), and shrinkage factor. External validation (one center, 2009–11).	**Complicated CDI course** - Prolonged admission to the intensive care unit due to CDI. - Colectomy du to CDI - Death as a direct or indirect consequence of CDI	Age (years)			-3 to 11	ICU admission for management of CDI, after enrollment. Hypotension was replaced by mean arterial pressure ≤ 65 mmHg
		50–84	1.83 (0.68–4.97)	1		
		≥ 85	4.96 (1.40–17.60)	3		
		CDI diagnosed in ICU	7.03 (2.02–24.40)	3		
		Recent abdominal surgery	0.23 (0.07–0.73)	-3		
		Hypotension	3.25 (1.53–6.91)	2		
		Diarrhea as a reason for admission	3.27 (1.57–6.80)	2		
**van der Wilden et al. 2013 [27]** Prospective cohort: inpatients, one center (2010–12), USA. - Follow-up: not reported. - Multivariable logistic regression. - Validation: internal	**Fulminant CD colitis** Systemic toxic effect resulting in: - ICU admission - Urgent colectomy - Death	Risk scoring system (RSS)			1 to 16	Cardiorespiratory failure considered at time of enrollment as need for vasopressors, mechanical ventilation or mean arterial pressure < 60 mmHg. Abdominal tenderness as pain during physical examination graded by the visual analog score (0–10)
		Age >70 years	3.80 (1.14–13.68)	2		
		WBC ≥20 or ≤2 × 10^9^/L	1.81 (0.54–6.05)	1		
		Cardiorespiratory failure^c^	285 (24.0–21,491)	7		
		Diffuse abdominal tenderness	189 (27.0–8,429)	6		
**Shivashankar et al. 2013 [30]** Retrospective cohort: inpatients, one center (2007–10), USA. - Follow-up: outcomes within 30 days of CDI diagnosis. - Multivariable logistic regression. - Validation: internal	**Severe-complicated CDI** - Admission to the ICU - Colectomy - Death	Age, 10 years increase	1.10 (1.0–1.10)	1^d^	Continuous	Creatinine increase > 1.5 × baseline values, adjusted for age and gender in non-black patients, excluding patients with chronic kidney disease and dialysis^e^. Histamine-2 blocker or PPI use within two months of CDI diagnosis.
		WBC ≥ 15 × 10^9^/L	2.20 (1.70–2.90)	0.81^d^		
		Narcotic use^d^	2.10 (1.50–3.00)	0.77^d^		
		H2-RAor PPI use^d^	1.80 (1.30–2.60)	0.63^d^		
		Creatinine ratio > 1.5	1.60 (1.30–2.10)	0.52^d^		
**Prediction of mortality**						
**Kassam et al. 2016 [29]** Retrospective cohort: Administrative databases, CDI-associated hospitalizations (2011 Nationwide Inpatient Sample), USA. - Follow-up: not reported. - Multivariable logistic regression. - Validation: internal and external, CDI hospitalizations in 2010 NIS, discrimination.	In-hospital mortality	Age (years)			-1 to 19	Acute kidney failure replaced by creatinine increase > 1.5 x baseline values, adjusted for age and gender in non-black patients, excluding patients with chronic kidney disease and dialysis^d^
		41–60	1.51 (1.24–1.82)	2		
		61–80	2.51 (2.06–3.06)	3		
		81–100	4.112 (3.39–4.99)	4		
		Critical care/ICU admission	5.29 (4.85–5.77)	5		
		Acute renal failure	2.93 (2.76–3.13)	3		
		Diabetes	0.83 (0.77–0.88)	-1		
		Serious comorbidities				
		Cardiopulmonary disease	1.46 (1.38–1.56)	1		
		Liver disease	2.00 (1.78–2.25)	2		
		IBD	1.72 (1.49–1.99)	2		
		Malignancy	1.89 (1.74–2.05)	2		
**Butt et al. 2013 [28]** Retrospective cohort: Inpatients, one center (2007–09), UK. - Follow-up: 30 days. - Classification tree. - Validation: internal and external, 158 patients in another setting (2006–07)	All-cause mortality	Serum albumin ≤ 24.5 g/L	0.80 (0.70–0.91)/ 0.84 (0.75–0.95)^f^	1	0–3	All-cause 30-day mortality
		CRP > 228 mg/L	1.01 (1.01–1.02)/ (0.99–1.02)^f^	1		
		WBC > 12 x 10^9^/L or respiratory rate > 17/min	WBC: 1.05 (1.01–1.09)/ 1.01 (0.95–1.07)^f^ Resp. rate: 1.22 (1.07–1.40)/1.19 (1.05–1.34)^f^	1		
**Archbald-Pannone et al. 2015 [31]** Prospective cohort: Inpatients, one center (2010–11), USA. - Follow-up: 30 days. - Wilcoxon rank sum test. - Validation: internal	Mortality attributed to CDI within 30 days of diagnosis All-cause death according to a correspondence with the author	Charlson score	Not reported, points attributed according to rescaled adjusted coefficients	2^g^	Continuous	Delirium according to NEECHAM confusion scale: [32]: 0–19 points = moderate to severe confusion 20–24 points = mild or early development of delirium 25–30 points = not confused or normal function
		Ln(WBC)		3^g^		
		Ln(BUN)		5^g^		
		CDI diagnosed in ICU		5^g^		
		Delirium		11^g^		

Abbreviations: AOR, adjusted odds ratio; BUN, blood urea nitrogen; CI, confidence interval; CRP, C-reactive protein; H2-RA, histamine receptor blockers; IBD, inflammatory bowel disease; ICU, intensive care unit; PPI, proton pump inhibitors; WCC, white blood cell count.

^a^ Assigned points for each predictor, otherwise zero.

^b^ In Na et al. “The relatedness of ICU admission or death to CDI was determined by chart review performed by two independent physician investigators. All of the severe outcomes evaluated occurred during the same hospital admission when CDI was diagnosed”.

^c^ Cardiorespiratory failure defined as CDI-related requirement of vasopressor and/or mechanical ventilation.

^d^ Predictive model with age as continuous variable, other variables dichotomized (0 = no, 1 = yes). Narcotic and antacids use were considered 7 days prior and 30 days after CDI diagnosis. Individual predictive score was computed with the formula: score = -3.07 + 0.01(age) + 0.81(WBC ≥ 15×1 0^9^) + 0.77(Narcotic use) + 0.63 (H2 blocker/PPI use) + 0.52(creatinine). Probability of severe-complicated CDI = 1/(1+e^(-score)^).

^e^ Ethnical origins of included patients was not available. The expected normal renal function of Caucasian patients was used as they represents 79% of the Canadian population.

^f^ A multinomial logistic regression was used with the outcome classified as CDI-related mortality, Non-CDI related mortality, or survival. aHR are shown for each predictor associated with CDI-related mortality/Non-CDI related.

^g^ A factor used to multiply the continuous or logarithmic value of each predictor.

A logistic regression was conducted for each CPR, and the following standard performance parameters with 95% confidence intervals (CI) were assessed [7]: sensitivity, specificity, positive and negative predictive values (PPV and NPV), positive and negative likelihood ratios (LR), and overall accuracy. Discrimination (the ability of a CPR to distinguish high-risk from low-risk patients) was assessed by the area under the receiver operating characteristic curve (AUC-ROC). Calibration (the extent of agreement between predicted probabilities by a CPR and the observed occurrence of an outcome) was assessed graphically and by Brier score or the mean absolute calibration error (MACE; %), where absolute values of deviance between observed and predicted probability were averaged [12]. Lower MACE values reflect greater precision of the predicted probability. Statistical analyses were performed using SAS 9.4 (SAS Institute Inc., Cary, North Carolina).

## Results

**Fig 1:**
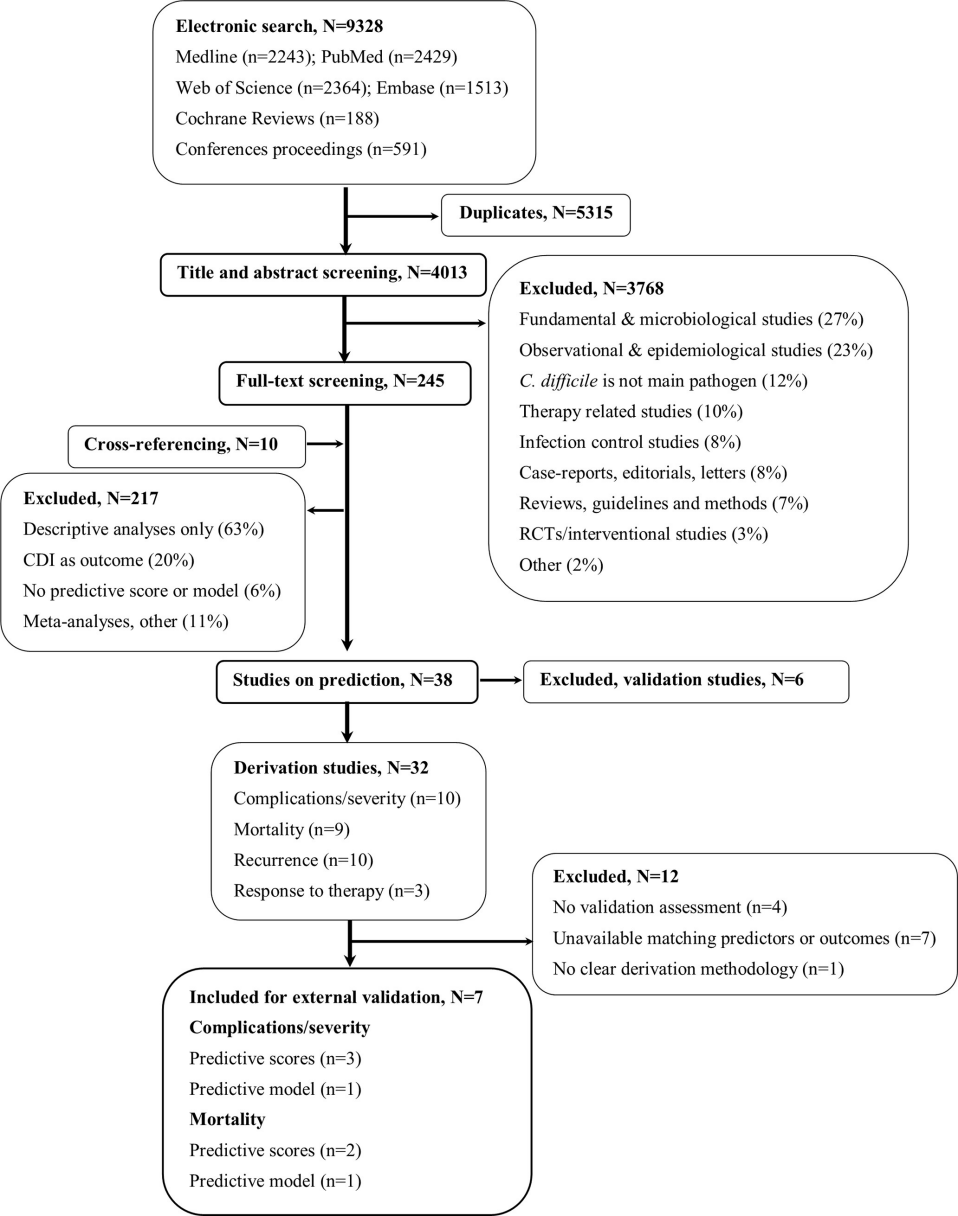
Flowchart of included and excluded publications (systematic review and update).

The systematic review flow-chart is shown in Fig 1. Overall, 32 studies with a derivation of a CPR for CDI unfavorable outcomes were identified. Among these, ten aimed to predict cCDI and nine aimed to predict CDI-associated mortality. We excluded four studies that did not report clear estimates of internal validity [13–16], seven studies for which important variables or outcomes were not available in our cohort [17–23], and one study that reported very limited information on derivation methodology [24]. We assessed the external validity of three scores predictive for cCDI [25–27] and two for mortality [28, 29], and one predictive model for each outcome, respectively [30, 31].

The characteristics of the included CPRs and relative modifications are shown in Table 1. Four studies used a prospective design for derivation [25–27, 31] and one was performed in multiple centers [26]. The duration of follow-up was reported in two studies [26, 31]. The derivation sample size was relatively small (n = 213 to 746 patients), except in the Kassam et al. [29] study, where data from an administrative database of CDI-associated hospitalizations were used (Table 1 and S2 Table). Complications of CDI were mainly defined by ICU admission, colectomy, or all-cause death. However, only Na et al. [25] restricted the outcome to CDI-attributable events. The distribution of outcomes varied greatly for cCDI and mortality, with rates of 6.4%-34%, and 8–21%, respectively (S1 Table). Most studies used multivariable logistic regression models to identify predictors. The complete data on predictors was reported in only two studies [26, 27] (S2 Table). The *C*. *difficile* strain (ribotype) was reported in only one study during an endemic period, without considering it among potential predictors [26]. Scoring points were attributed in proportion to risk estimates in two studies [28, 29] (Table 1). The AUC-ROC was the most frequently reported performance measure for internal validation (Tables 2 and 3).

**Table 2:** Performance of scores (95%CI) and models for prediction of CDI complications in the external validation cohort.

Study	n; % outcome	Cut-off (n patients; %)	Observed outcome/ score n (%)	Sensitivity (%)	Specificity (%)	PPV (%)	NPV (%)	Positive LR	Negative LR	Accuracy (%)	AUC	MACE (%)	OR for 1-point increase in score
**Na et al. [25]**	**n = 1318; 7.81**	**0–1 pts** (1113; 84.5)	56 (5.0)	100	-	7.8 (6.4–9.3)	-	1.0	-	-	0.66 (0.61–0.73)	13.6	2.8 (2.1–3.7)
		**2–3 pts** (205; 15.6)	47 (22.9)	45.6 (36.0–55.2)	87.0 (85.1–88.9)	22.9 (17.2–28.7)	95.0 (93.7–96.3)	3.5 (3.3–3.7)	0.63 (0.60–0.65)	83.8 (81.7–85.7)			
**Hensgens et al. [26]**	**n = 1338; 14.95**	**< 0 pts** (118; 8.8)	5 (4.2)	90.5 (86.4–94.6)	21.8 (19.4–24.2)	16.9 (14.7–19.1)	92.9 (89.8–96.0)	1.15 (1.15–1.16)	0.44 (0.38–0.50)	32.1 (29.6–34.6)	0.63 (0.59–0.68)	24.5	1.3 (1.2–1.4)
		**0–1 pt** (709; 53.0)	87 (12.3)	54.0 (47.1–60.9)	64.6 (61.8–67.4)	21.1 (17.6–24.7)	88.9 (86.7–91.0)	1.50 (1.49–1.56)	0.71 (0.69–0.73)	63.0 (60.4–65.6)			
		**2–3 pts** (344; 25.7)	57 (16.6)	53.0 (46.1–59.9)	66.9 (64.1–69.6)	21.9 (18.3–25.6)	89.0 (86.9–91.1)	1.60 (1.57–1.64)	0.70 (0.69–0.72)	64.8 (62.2–67.3)			
		**≥ 4 pts** (167; 12.5)	51 (30.5)	25.5 (19.5–31.5)	89.8 (88.0–91.6)	30.5 (23.6–37.5)	87.3 (85.4–89.2)	2.50 (2.2–2.8)	0.83 (0.81–0.84)	80.2 (78.0–82.2)			
**van der Wilden et al. [27]**	**All-cause 30-day death; all-cause ICU admission; hemi/colectomy n = 1330; 18.63**	**< 6 pts** (714; 53.7)	128 (17.9)	100	-	18.6 (16.5–20.7)	-	1.0	-	-	0.57 (0.53–0.61)	11.5	1.09 (1.04–1.13)
		**≥ 6 pts** (616; 46.3)	119 (19.3)	48.2 (41.9–54.4)	54.4 (51.1–57.1)	19.3 (16.2–22.4)	82.1 (79.3–84.9)	1.05 (1.03–1.07)	0.96 (0.95–0.97)	53.0 (50.3–55.7)			
	**CDI attributable or cause of 30-day death; CDI attributable ICU admission; hemi/colectomy n = 1321; 6.81**	**< 6 pts** (709; 53.7)	35 (4.9)	100	-	6.8 (5.4–8.2)	-	1.0	-	-	0.67 (0.61–0.73)	12.1	1.2 (1.15–1.3)
		**≥ 6 pts** (612; 46.3)	55 (9.0)	61.1 (51.0–71.2)	54.7 (52.0–57.5)	9.0 (6.7–11.2)	95.1 (93.5–96.7)	1.35 (1.32–1.39)	0.71 (0.67–0.75)	55.2 (52.5–57.8)			
**Shivashankar et al. [30]**	**n = 1026; 17.25** Min score -2.9, max 0.61 Min prob. 0.05, max 0.65	**≤ -2.0** (307; 51.1)		85.9 (80.7–91.0)	27.8 (24.8–30.8)	19.9 (17.0–22.7)	90.4 (86.8–94.0)	1.19 (1.18–1.20)	0.51 (0.46–0.56)	37.8 (34.9–40.8)	0.66 (0.62–0.71)	26.9	2.8 (2.1–3.8)
		**-1.6**		58.7 (51.5–66.0)	60.9 (57.6–64.2)	23.8 (19.8–27.8)	87.6 (85.0–90.3)	1.50 (1.47–1.53)	0.68 (0.66–0.70)	60.5 (57.5–63.5)			
		**-1.4**		51.9 (44.6–59.3)	70.4 (67.4–73.5)	26.8 (22.1–31.5)	87.6 (85.1–90.0)	1.76 (1.71–1.81)	0.68 (0.66–0.70)	67.2 (64.3–70.1)			
		**-1.2**		45.7 (38.4–53.1)	75.4 (72.5–78.3)	27.9 (22.8–33.1)	87.0 (84.5–89.4)	2.09 (1.98–2.20)	0.74 (0.73–0.76)	73.7 (70.9–76.3)			
		**-1.1**	Probability ~25%	35.6 (28.5–42.6)	84.1 (81.6–86.6)	31.8 (25.3–38.3)	86.2 (83.9–88.6)	2.34 (2.09–2.40)	0.77 (0.75–0.78)	75.7 (73.0–78.3)			
		**-0.8**		21.5 (15.4–27.5)	94.5 (92.9–96.0)	44.7 (34.1–55.3)	85.2 (83.0–87.5)	3.88 (3.08–4.88)	0.83 (0.82–0.84)	81.9 (79.4–84.1)			
		**-0.4**		9.0 (4.8–13.3)	97.2 (96.1–98.3)	40.0 (24.8–55.2)	83.7 (81.4–86.0)	3.20 (0.86–11.90)	0.94 (0.92–0.95)	82.0 (79.5–84.2)			

AUC, area under the ROC curve; LR, likelihood ratio; MACE, mean absolute calibration error; NR, not reported; OR, odds ratio estimated with a univariable logistic regression.

**Table 3:** Performance (95%CI) of scores and models for prediction of mortality in the external validation cohort.

Study	n; % outcome	Cut-off (n patients; %)	Observed outcome/ score n (%)	Sensitivity (%)	Specificity (%)	PPV (%)	NPV (%)	Positive LR	Negative LR	Accuracy %	AUC	MACE (%)	OR for 1-point increase in score
**Kassam et al. [29]**	**n = 1045; 17.61**	**-1-0 pts** (44; 4.2)	3 (6.8)	100	0.4	17.7 (15.3–20.0)	100	1.0	-		0.66 (0.62–0.71)	27.18	1.3 (1.2–1.4)
		**1–5 pts** (742; 71.0)	100 (13.5)	62.0 (54.9–69.0)	59.0 (55.7–62.3)	24.4 (20.5–28.3)	87.9 (85.2–90.5)	1.51 (1.49–1.54)	0.64 (0.62–0.66)	59.5 (56.5–62.5)			
		**6–10 pts** (225; 21.5)	64 (28.4)	44.0 (36.8–51.2)	79.3 (76.6–82.0)	31.3 (25.6–36.9)	86.9 (84.5–89.2)	2.71 (2.64–2.78)	0.55 (0.54–0.57)	75.2 (72.5–77.7)			
		**11–14 pts** (34; 3.3)	17 (50.0)	9.2 (5.1–13.4)	98.0 (97.1–98.9)	50.0 (33.2–66.8)	83.5 (81.2–85.8)	4.68 (1.34–16.30)	0.93 (0.92–0.94)	82.4 (80.0–84.6)			
**Butt et al. [28]**	**n = 933; 12.11**	**0 pts** (494; 52.9)	32 (6.5)	100	-	12.1 (10.0–14.2)	-	-	-	12.1 (10.2–14.4)	0.69 (0.63–0.75)	19.39	2.7 (2.1–3.4)
		**1 pts** (314; 33.6)	38 (12.1)	71.7 (63.4–80.0)	56.3 (52.9–59.7)	18.4 (14.4–22.1)	93.5 (91.3–95.7)	1.64 (1.62–1.67)	0.50 (0.47–0.54)	58.2 (55.0–61.3)			
		**2 pts** (111; 11.9)	33 (29.7)	38.0 (29.1–47.0)	90.0 (87.9–92.0)	34.4 (26.1–42.7)	91.3 (89.4–93.3)	3.81 (3.45–4.20)	0.69 (0.67–0.71)	83.7 (81.2–85.9)			
		**3 pts** (14; 1.5)	10 (71.4)	8.8 (3.6–14.1)	99.5 (99.0–100.0)	71.4 (47.8–95.1)	88.8 (86.7–90.8)	18.14 (1.48–223)	0.92 (0.89–0.93)	88.5 (86.3–90.4)			
	**Score without respiratory rate**^**a**^ **n = 940; 12.45**	**0 pts** (474; 50.4)	32 (6.7)	100	-	12.4 (10.3–14.6)	-			12.4 (10.5–14.7)	0.69 (0.64–0.75)	19.85	2.6 (2.1–3.3)
		**1 pts** (323; 34.4)	36 (11.2)	72.6 (64.6–80.7)	53.7 (50.3–57.1)	18.2 (14.7–21.7)	93.2 (91.0–95.5)	1.57 (1.55–1.59)	0.51 (0.48–0.54)	56.1 (52.9–59.2)			
		**2 pts** (126; 13.4)	38 (30.2)	41.9 (32.9–50.8)	88.6 (86.4–90.7)	34.3 (26.5–42.0)	91.5 (89.5–93.4)	3.67 (3.40–3.96)	0.66 (0.64–0.68)	82.8 (80.2–85.0)			
		**3 pts** (17; 1.8)	11 (64.7)	9.4 (4.1–14.7)	99.3 (98.7–99.8)	64.7 (42.0–87.4)	88.5 (86.5–90.6)	12.90 (1.67–99.54)	0.91 (0.89–0.93)	88.1 (85.9–90.0)			
**Archbald-Pannone et al. [31]**	**n = 1235; 12.2**3 Range -1.8–87.2 pts; Median 24.9; IQR: 18.2–34.1	**< 10 pts** (48; 3.9)	0	100	-	12.2 (10.4–14.1)	-	1.0	-	12.2 (10.5–14.2)	Expected: 0.770^b^ /Observed: 0.74 (0.69–0.78)	19.46	1.05 (1.03–1.06)
		**10–20 pts** (350; 28.3)	14 (4.0)	100	4.4 (3.2–5.6)	12.7 (10.8–14.6)	100	1.05 (1.04–1.048)	-	16.1 (14.2–18.3)			
		**20–30 pts** (411; 33.3)	36 (8.8)	90.7 (86.1–95.3)	35.4 (32.6–38.3)	16.4 (13.9–18.9)	96.5 (94.7–98.3)	1.40 (1.40–1.41)	0.26 (0.22–0.30)	42.2 (39.5–45.0)			
		**30–40 pts** (233; 18.9)	47 (20.2)	66.9 (59.4–74.4)	70.0 (67.3–72.7)	23.7 (19.7–27.7)	93.8 (92.2–95.5)	2.11 (2.08–2.14)	0.48 (0.47–0.50)	68.1 (65.4–70.7)			
		**40–50 pts** (96; 7.8)	19 (19.8)	35.8 (28.1–43.4)	87.2 (85.2–89.2)	28.0 (21.6–34.3)	90.7 (88.9–92.5)	2.79 (2.58–3.02)	0.74 (0.72–0.75)	80.9 (78.6–83.0)			
		**50–60 pts** (54; 4.4)	15 (27.8)	23.2 (16.4–29.9)	94.3 (92.9–95.7)	36.1 (26.5–45.6)	89.8 (88.0–91.6)	4.05 (3.26–5.04)	0.81 (0.80–0.83)	85.6 (83.5–87.4)			
		**60–70 pts** (30; 2.4)	10 (33.3)	13.2 (7.8–18.6)	97.9 (97.0–98.7)	46.5 (31.6–61.4)	89.0 (87.2–90.8)	6.24 (3.02–12.92)	0.88 (0.87–0.90)	87.5 (85.6–89.3)			
		**≥ 70 pts** (13; 1.1)	10 (76.9)	6.6 (2.7–10.6)	99.7 (99.4–100.0)	76.9 (54.0–99.8)	88.5 (86.7–90.2)	23.93 (0.78–29.30)	0.94 (0.92–0.95)	88.5 (86.4–90.0)			

AUC, area under the ROC curve; LR, likelihood ratio; MACE, mean absolute calibration error; NR, not reported; OR, odds ratio estimated with a univariable logistic regression.

^a^ An external validation was performed on data from the study of Bhangu et al. 2010 [16] omitting respiratory rate. The modified score showed an AUC of 0.754 (95%CI, 0.67–0.84) in the derivation cohort (n = 244), and of 0.704 (95%CI, 0.62–0.79) in the validation cohort (n = 154).

^b^ As reported in study.

### External validation

Sample sizes for data that were used in external validation of each CPR varied from 933–1338 patients in each sub-cohort (S1 Table), without major differences from the overall (or full) external validation cohort [11]. Most cases were older adults (median age 71), had at least one chronic underlying illness (88%), had received at least one antimicrobial agent (87%) within two months preceding enrollment, had received at least one acid suppression medication (66%), had undergone surgery (38%), and were immunocompromised (29%). At enrollment, CDI was the initial episode in 86% of patients and was healthcare-associated in 90%. Strain ribotype was obtained for 922 patients; ribotype 027 was found in 52% of them. Metronidazole was the initial treatment in 81% of patients, vancomycin in 8%, and a combination of these drugs in 6.5%. During the 30-day follow-up period, 3% of patients were admitted to ICU for complications of CDI, and 12% died from all causes. Only 4% of deaths were deemed attributable to CDI.

### CPRs for cCDI

For all four studies that were assessed, the observed cCDI outcomes were lower in each validation sub-cohort than in the respective derivation cohorts (Table 1, S3 Table). Calibration curves are shown in S1 Fig. For a score ≥2 points in Na et al. [25], sensitivity was lower in our cohort (46%) compared to both the internal assessment (62%) and the external validation cohorts reported in the publication (53%). The external validation was conducted on a cohort of combined patients from two centers in two different countries (n = 345) (S3 Table). The PPV of the score decreased by two-fold in our validation (23% vs 44%). Specificity, the NPV, both LRs, and diagnostic accuracy were similar in the derivation and the reported external validation cohorts, but were higher in ours, although the discrimination level was only 66% (AUC) and the calibration error was 14% (Table 2).

Performance parameters decreased for all cut-offs of Hensgens’ score [26] in our validation model and outcomes were more frequently observed in patients with low scores (11%, n = 92/827 vs. 3%, n = 7/219 with a score ≤ 1 point). We obtained lower sensitivity for a score of ≥4 points than both reported internal and external validations (25% vs. 43%), but with comparable specificity (90%). Both discrimination and calibration were low in the external validation cohort (AUC = 0.63 and MACE = 25%).

In the study by van der Wilden et al. [27], attributable and all-cause events were not differentiated. We conducted validation using all-cause and CDI-attributable ICU admissions and death (Table 2). In both cases, all observed parameters were significantly lower than in the derivation cohort (S3 Table), where a score ≥6 points was extremely sensitive (98% vs. 48% and 61%, respectively) but less specific (88% vs. 54%). This score showed perfect discrimination in the derivation cohort (0.98) that dramatically decreased in external validation (0.6–0.7), with 12% error in calibration. Diffuse abdominal tenderness, a criterion generating 6 out of 16 points in this score, was 2.6-fold more frequent in our cohort (S2 Table), and 46% of patients had high scores. Despite a very low PPV (9% vs. 19%), using CDI-attributable vs. all-cause events to define cCDI led to higher LR+ (1.35 vs. 1.05) values, slightly higher overall accuracy (55%), and better discrimination (0.67 vs. 0.57) and calibration (MACE 12% vs. 30%).

The optimal cut-off point (score = -1.1) for the Shivashankar et al. [30] model corresponded to a 25% probability of developing cCDI in both the derivation and external validations. However, this score had 80% sensitivity and 46% specificity in the derivation cohort (S3 Table), vs. 36% and 84%, respectively in the validation cohort. We observed 50% of the frequency of the outcome (17% vs. 34%), and the AUC was similar in both cohorts (0.70). Although each increasing point in the score was associated with a 3-fold increased risk of cCDI (crude OR = 2.9), this model showed 27% error in calibration. However, the sensitivity steadily decreased, while specificity increased with the scoring points. PPV reached 45% in the external validation cohort, but was not reported in the derivation study (Table 2).

### CPRs for mortality

Calibration plots are shown in S2 Fig. Despite the much larger sample size, in-hospital mortality was about two-fold higher in the external validation sub-cohort (18% vs. 8%) than in the derivation cohort used in Kassam et al. [29]. Lower scores were more frequently observed in the validation sub-cohort, with 75% of patients having ≤ 6 points with a maximum observed of 14 points (Table 3). These frequencies were not reported in the derivation study (S4 Table). Only 50% of patients with 11–14 points experienced the outcome (n = 17), leading to a low sensitivity and PPV (9% and 50% respectively). Both discrimination and calibration were lower in the validation sub-cohort (AUC-ROC = 0.66; MACE = 27%) despite a 30% increased risk of mortality associated with each point increase in the score (crude OR = 1.28).

The study of Butt et al. [28] had higher all-cause mortality in the derivation cohort than in the validation sub-cohort (20% vs. 12%). Only 7% of patients had ≥2 points in the derivation cohort (n = 18/244), among whom 72% had died (n = 13), while 19% of patients assigned ≥2 points (14%, n = 125/933) in the validation sub-cohort died. When the respiratory rate criterion was dropped, as in the original study, the sensitivity for a score ≥ 2 points increased to 42%, with a moderate PPV (34%), and without any significant changes in the other parameters. The AUC value was the only reported parameter (S4 Table), and was similar in the external validation cohort. Independent of the respiratory rate criterion, the calibration error for this score was about 20%.

For the Archbald-Pannone et al. [31] model, a one-point increase was associated with a 5% increased risk of all-cause 30-day mortality in the validation sub-cohort (crude OR = 1.05), compared to 11% in the derivation cohort (S4 Table). Levels of WBCs and blood urea nitrogen (BUN) were lower in the validation sub-cohort (S2 Table). Overall, 14% of patients were assigned scores of ≥ 50 points in the derivation cohort, among whom 42% died (n = 22), vs. only 8% of patients with 36% mortality (n = 35) in the validation sub-cohort. The model showed significant increases in PPV with increasing points (77% for high scores), but a much lower sensitivity (7%). The AUC was of 0.74, which was lower than the internal validation (0.80), but comparable to the expected value of 0.77, with 19% error in calibration.

## Discussion

In this study, we validated four prediction rules for CDI complications and three for CDI associated-mortality using a cohort with a low incidence of outcomes (8% cCDI and 12% mortality), in which more than half of cases were attributed to the R027 strain. External validation is a mandatory step in taking a prediction rule from development to clinical integration, as it addresses the transportability of the score. This study aimed to evaluate published scores performance in a large, multicentre, and prospective cohort, and included patients with demographic and clinical characteristics of typical CDI patients [11]. All predictor and outcome definitions in each included study were closely reproduced, with the exception of a few studies in which we adapted the original definitions to fit the variables in our cohort. All included scores and models showed a decrease in the performance of their predictive potential in our cohort, even when the results from the internal validation were promising [27]. Calibration, as defined by the mean error between observed and expected outcomes, ranged from 12 to 30%.

Using the same cut-offs as in derivation studies, the CPRs for cCDI showed sensitivities ranging from 25% to 61%, specificities from 54% to 90%, PPV from 9% to 31%, and NPV from 82% to 95% in our validation cohort. The overall accuracy and AUC values were low, ranging from 53% to 84% and from 0.57 to 0.67, respectively. However, a decrease in performance is not unusual in the external validation of CPRs [7]. For example, the scores of Na and Hensgens [25, 26] performed similarly (AUC = 0.54 and 0.68 respectively) in a second validation cohort of 148 patients during an outbreak of R027 strains [33]. The effect of strain on CDI outcome is still controversial in observational studies [34], and we did not find any significant association with cCDI in our cohort [11], despite the high frequency of R027. Only one of the included studies reported the frequency of ribotypes, and it was not considered a potential predictor [26]. Similar performance was observed for mortality, with sensitivities ranging from 4% to 55%, specificities from 71% to 78%, PPVs from 25% to 31%, and NPVs from 87% to 92%. Overall accuracy ranged from 73% to 82% and the AUC was also moderate, ranging from 0.66 to 0.69.

In the time since most of the included CPRs were published, methodological standards [35] and quality assessment criteria [36] based on predictive modelling and prognostic studies have been released. Accordingly, important methodological limitations of the included CPRs could have affected their performance in derivation as well as in the external settings, as most of the CPRs in this study were derived from small samples sizes, and in different settings. Consequently, it is not surprising to find heterogeneity between the different variables identified as predictors. There were no common predictors in studies predicting mortality, and a limited number of common predictors in studies predicting cCDI, except for older age (increased WBC was a predictor in three studies [25, 27, 30], and increased serum creatinine and hypotension were each common predictors in two studies [25–27, 30]). In contrast, the CARDS score characterized by Kassam et al. [29], which was derived from a medico-administrative database, allowed for a large sample size. However, this study also reported less detailed data and used ICD-9 discharge codes for case definitions. A more recent score was derived using the same criteria as CARDS to predict 30-day mortality following complete colectomy [23]. This score was not included for external validation, due to a very low outcome occurrence in our cohort (4 deaths in 18 patients who underwent a colectomy). In the study by Na et al. [25], each predictor was assigned one point despite the serum creatinine having a four-fold higher risk estimate, and WBC counts having a two-fold higher risk estimate, compared to age (OR, 8, 4, and 2.3 respectively). In the study by Shivashankar et al. [30], narcotics and antacids used during the seven days before CDI diagnosis and up to 30 days after diagnosis were considered predictors for cCDI. In the score of Butt et al. [28], low albumin levels were protective in the derivation study, but were assigned the same number of points as the other criteria. For each point increase in their continuous score, risk of all-cause death was two-fold higher than in-hospital mortality [29].

Differences in predictor frequencies between the derivation and the validation sub-cohorts might also have influenced the performance of the CRPs. In the van der Wilden study [27], diffuse abdominal tenderness was three times more frequent than in our cohort and shifted individual scores to higher levels. Also, in the Archbald-Pannone model [31], only 14% of the patients in the validation cohort presented with delirium, a predictor that was given 11 points. Using CDI-attributable unfavorable events in the van der Wilden score [27] led to better prediction than all-cause ICU admission and mortality in terms of discrimination, and decreased calibration from 30% to 12%.

Other studies have attempted external validation of indices for severe CDI course on much smaller sample sizes [33, 37–39]. While we identified relevant studies through a rigorous systematic review of the literature and included only studies with clear derivation methodology and at least one internal validation assessment. Only van Beurden et al. [33] used a standardized selection of studies.

Recent guidelines from the Infectious Diseases Society of America (IDSA) [40] highlight two models developed from the fidaxomicin vs. vancomycin clinical trial data, which identified factors that correlated with treatment failure and cure [41, 42]. These guidelines used expert opinions to define severe or fulminant CDI and underlined the need for a prospectively validated severity score. Many scores have been published throughout the years, but none have shown performance data sufficient for wide clinical use, which reflects the complexity of this task. In the future, other types of predictors should probably be considered and integrated into prediction models such as levels of toxins and measures of immunity, frailty, and bacterial strains.

## Conclusion

Clinical prediction rules for cCDI and CDI mortality showed moderate performances in an external validation cohort that had a low rate of measured outcomes and a high proportion of R027 strains. The methodological limits of the original studies and the heterogeneity of the primary outcomes may have contributed to these suboptimal results. An accurate predictive tool is needed to help clinicians and researchers identify patients at risk for cCDI, and to direct the most effective therapies to these patients.

## Supporting information

S1 Fig Calibration plots for scores (95%CI) and models for prediction of CDI complications in the external validation cohort. (DOCX)

S2 Fig Calibration plots for scores (95%CI) and models for prediction of CDI mortality in the external validation cohort. (DOCX)

S1 Table Characteristics of patients in the original cohort and in data used for the external validation of each score or model. (DOCX)

S2 Table Frequencies of predictors in the derivation cohort and in data used for the external validation. (DOCX)

S3 Table Reported performance of scores (95%CI) and models for prediction of CDI complications. (DOCX)

S4 Table Reported performance (95%CI) of scores and models for prediction of mortality. (DOCX)
