# Supplementary material for: External validation of clinical prediction rules for complications and mortality following Clostridioides difficile infection
Source: PLoS One. 2019 Dec 17;14(12):e0226672. doi: 10.1371/journal.pone.0226672 (PMC6917260; doi:10.1371/journal.pone.0226672)
Supplement: S1 Fig — (DOCX) [file pone.0226672.s001.docx]

**S1 Fig- Calibration plots for scores (95%CI) and models for prediction of CDI complications in the external validation cohort**

**Na et al. 2015**


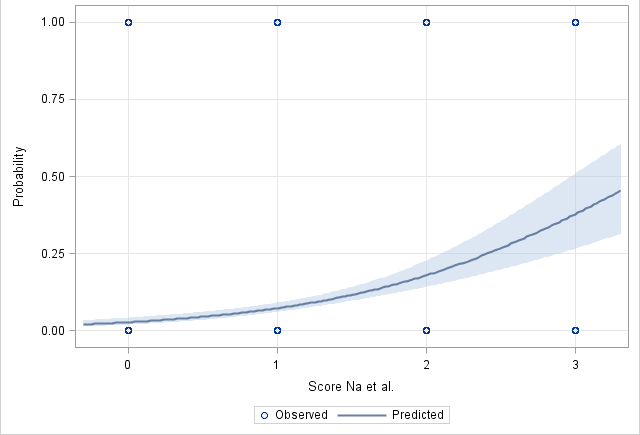


**Hensgens et al. 2014**

**
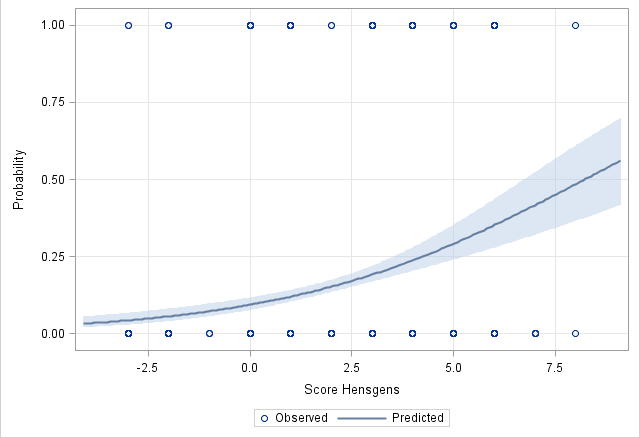
**

**Shivashankar et al. 2013**


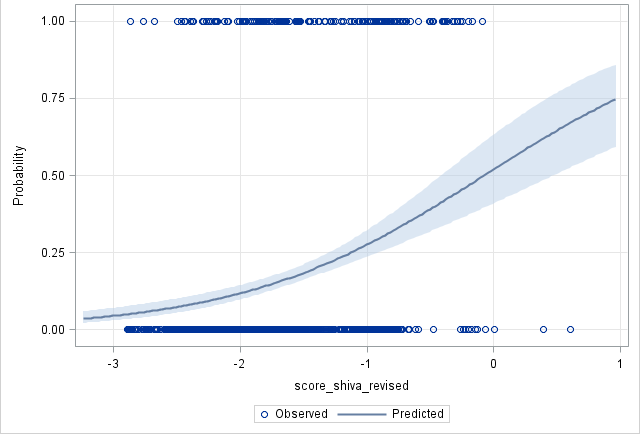


**van der Wilden et al. 2013**


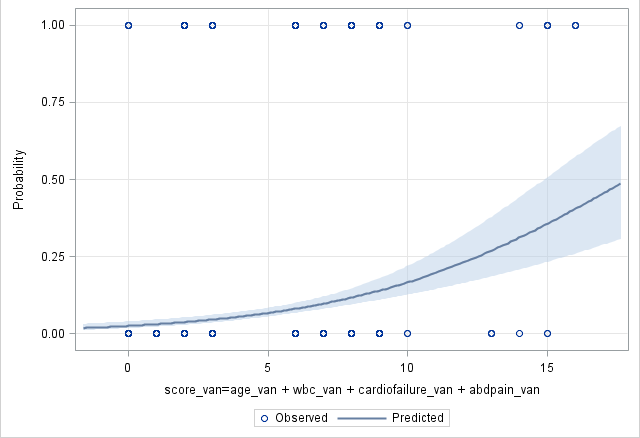


Score van der Wilden
