# Supplementary material for: External validation of clinical prediction rules for complications and mortality following Clostridioides difficile infection
Source: PLoS One. 2019 Dec 17;14(12):e0226672. doi: 10.1371/journal.pone.0226672 (PMC6917260; doi:10.1371/journal.pone.0226672)

**S2 Fig- Calibration plots for scores (95%CI) and models for prediction of CDI mortality in the external validation cohort**

**Butt et al. 2013**

**
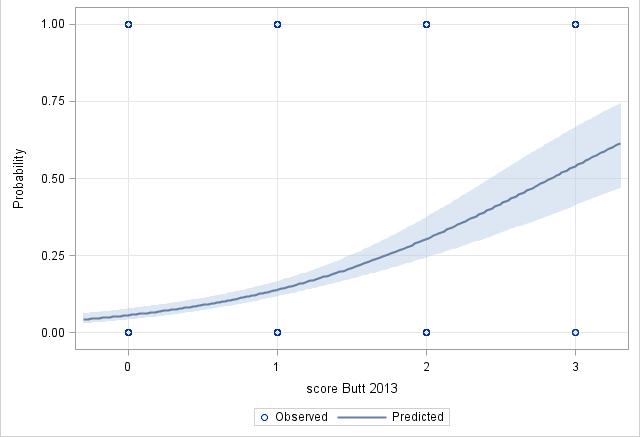
**

**Butt et al. 2013- Without respiratory rate**


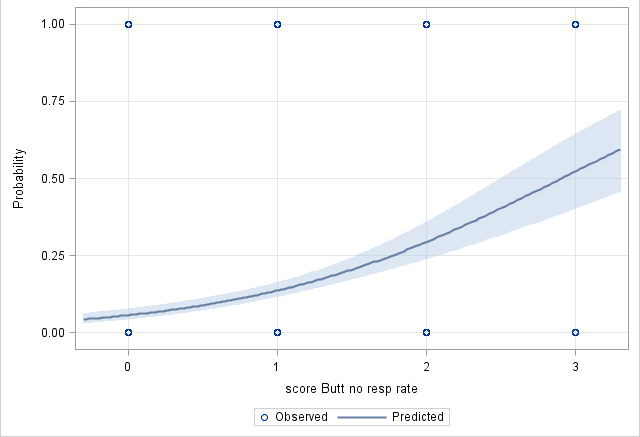


**Archbald-Pannone et al. 2015**


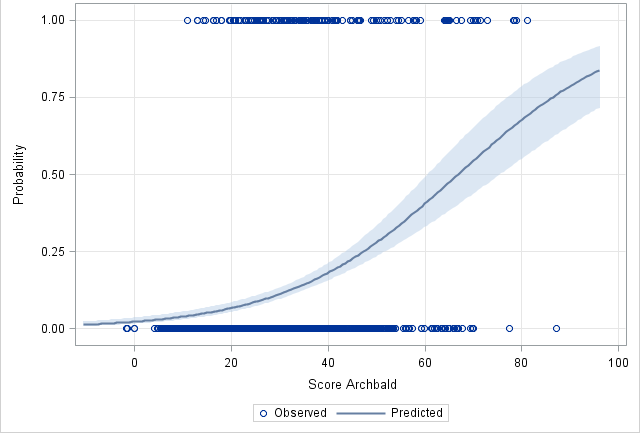


**Kassam et al. 2016**


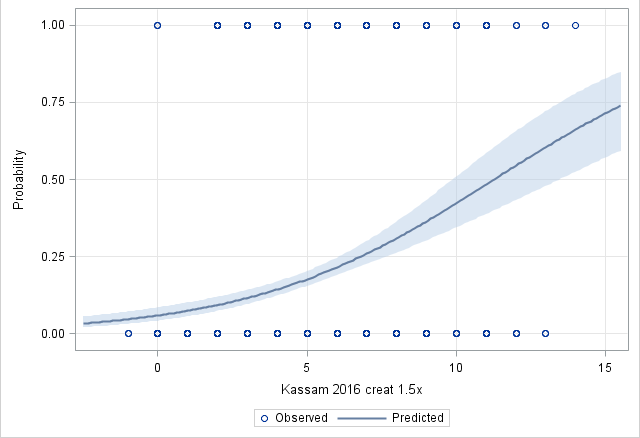

Supplement: S2 Fig — (DOCX) [file pone.0226672.s002.docx]
