# Supplementary material for: External validation of clinical prediction rules for complications and mortality following Clostridioides difficile infection
Source: PLoS One. 2019 Dec 17;14(12):e0226672. doi: 10.1371/journal.pone.0226672 (PMC6917260; doi:10.1371/journal.pone.0226672)
Supplement: S1 Table — (DOCX) [file pone.0226672.s003.docx]

**S1 Table. Characteristics of patients in the original cohort and in data used for the external validation of each score or model**

**Results are reported as n (%)**

| **Variable** | **Original CDI cohort [9]**  **(n = 1380)** | **Na et al. [24]**  **(n = 1318)** | **Hensgens et al. [25]**  **(n = 1338)** | **van der Wilden et al. [26]**  **(n = 1321)** | **Shivashankar et al. [29]**  **(n = 1026)** | **Kassam et al. [28]**  **(n = 1045)** | **Butt et al. [27]**  **(n = 933)** | **Archbald-Pannone**  **et al. [30] (n = 1235)** |
| --- | --- | --- | --- | --- | --- | --- | --- | --- |
| **Age (median; IQR)** | 71; 58-80 | 71; 58-80 | 71.2; 58-80.4 | 70.9; 58-80 | 70; 56.8-79.9 | 70; 56.8-79.9 | 70; 57-79.4 | 71; 57.7-80 |
| **Sex** |  |  |  |  |  |  |  |  |
| Female | 665 (48.2) | 639 (48.5) | 641 (47.9) | 636 (48.1) | 500 (48.7) | 510 (48.8) | 446 (47.8) | 594 (48.1) |
| Male | 715 (51.8) | 679 (51.5) | 697 (52.1) | 685 (51.9) | 526 (51.3) | 535 (51.2) | 487 (52.2) | 641 (51.9) |
| **Charlson comorbidity index** |  |  |  |  |  |  |  |  |
| 0-3 | 747 (54.1) | 711 (54.0) | 721 (53.9) | 710 (53.8) | 639 (62.3) | 650 (62.2) | 499 (53.5) | 667 (54.0) |
| 4-6 | 399 (28.9) | 381 (28.9) | 391 (29.2) | 383 (29.0) | 242 (23.6) | 248 (23.7) | 267 (28.6) | 353 (28.6) |
| ≥7 | 234 (17.0) | 226 (17.2) | 226 (16.9) | 228 (17.3) | 145 (14.1) | 147 (14.1) | 167 (17.9) | 215 (17.4) |
| **Antimicrobial exposure^a^ (within 2 mo)** | 1201 (87.0) | 1150 (87.3) | 1168 (87.3) | 1152 (87.2) | 897 (87.4) | 910 (87.1) | 808 (86.6) | 1071 (86.7) |
| Fluoroquinolones | 707 (51.0) | 677 (51.4) | 682 (51.0) | 674 (51.1) | 519 (50.6) | 525 (50.2) | 482 (51.7) | 627 (50.8) |
| Cephalosporins | 694 (50.0) | 667 (50.7) | 677 (50.6) | 670 (50.8) | 523 (51.0) | 529 (50.6) | 455 (48.8) | 619 (50.2) |
| Carboxy/ureidopenicillins | 274 (20.0) | 260 (19.7) | 266 (19.9) | 263 (19.9) | 198 (19.3) | 199 (19.0) | 189 (20.3) | 246 (19.9) |
| Macrolides/clindamycin | 251 (18.0) | 243 (18.4) | 247 (18.5) | 245 (18.6) | 192 (18.7) | 191 (18.3) | 165 (17.7) | 232 (18.8) |
| Antistaphylococcal/aminopenicillins | 225 (16.3) | 215 (16.3) | 218 (16.3) | 215 (16.3) | 165 (16.1) | 167 (16.0) | 150 (16.1) | 193 (15.6) |
| **Acid suppression agents (within 2 mo)** | 917 (66.4) | 880 (66.8) | 896 (67.0) | 886 (67.1) | 662 (64.5) | 666 (63.7) | 624 (66.9) | 826 (66.9) |
| PPI | 577 (42.0) | 554 (42.0) | 561 (41.9) | 553 (41.9) | 390 (38.0) | 394 (37.7) | 409 (43.8) | 517 (41.9) |
| H2-RA | 191 (13.8) | 180 (13.7) | 187 (14.0) | 185 (14.0) | 156 (15.2) | 160 (15.3) | 115 (12.3) | 166 (13.4) |
| PPI + H2-RA | 148 (10.7) | 146 (11.1) | 148 (11.1) | 148 (11.2) | 116 (11.3) | 112 (10.7) | 100 (10.7) | 143 (11.6) |
| **Immunosuppression (within 6 mo)** | 401 (29.3) | 389 (29.7) | 391 (29.4) | 391 (29.8) | 300 (29.5) | 302 (29.1) | 285 (30.8) | 363 (29.6) |
| **Surgery (within 2 mo)** | 525 (38.0) | 510 (38.7) | 514 (38.4) | 513 (38.8) | 426 (41.5) | 424 (40.6) | 360 (38.6) | 471 (38.1) |
| **CDI diagnosis method** |  |  |  |  |  |  |  |  |
| Conventional toxins A+B EIA | 1001 (72.5) | 944 (71.6) | 960 (71.7) | 945 (71.6) | 753 (73.4) | 775 (74.2) | 634 (68.0) | 864 (70.0) |
| GDH + toxin A detection | 239 (17.3) | 235 (17.8) | 238 (17.8) | 236 (17.9) | 164 (16.0) | 163 (15.7) | 192 (20.7) | 232 (18.9) |
| Cytotoxicity assay | 83 (6.0) | 82 (6.2) | 83 (6.1) | 82 (6.2) | 65 (6.4) | 65 (6.2) | 69 (7.4) | 82 (6.7) |
| Rapid toxins A+B EIA | 52 (3.8) | 52 (3.9) | 52 (3.9) | 52 (3.9) | 39 (3.8) | 37 (3.6) | 34 (3.7) | 52 (4.2) |
| Endoscopy | 5 (0.4) | 5 (0.4) | 5 (0.4) | 5 (0.4) | 5 (0.5) | 5 (0.5) | 4 (0.4) | 5 (0.4) |
| **Origin of CDI** |  |  |  |  |  |  |  |  |
| Hospital onset-HCFA | 1126 (81.6) | 1074 (81.5) | 1093 (81.7) | 1079 (81.7) | 827 (80.6) | 842 (80.6) | 764 (81.9) | 1003 (81.2) |
| Community onset-HCFA | 111 (8.0) | 108 (8.2) | 109 (8.2) | 107 (8.1) | 87 (8.5) | 88 (8.4) | 71 (7.6) | 103 (8.3) |
| Community-acquired | 143 (10.4) | 136 (10.3) | 136 (10.2) | 135 (10.2) | 112 (10.9) | 115 (11.0) | 98 (10.5) | 129 (10.5) |
| **Primary CDI episode** | 1180 (85.8) | 1129 (86.0) | 1142 (85.7) | 1134 (86.2) | 893 (87.5) | 907 (87.2) | 796 (85.7) | 1057 (85.9) |
| **Ribotype** |  |  |  |  |  |  |  |  |
| R027 | 483 (35.0) | 458 (34.8) | 467 (34.9) | 456 (34.5) | 345 (33.6) | 352 (33.7) | 325 (34.8) | 429 (34.7) |
| Other | 439 (31.8) | 418 (31.7) | 423 (31.6) | 421 (31.9) | 339 (33.0) | 343 (32.8) | 287 (30.8) | 387 (31.3) |
| Unavailable | 458 (33.2) | 442 (33.5) | 448 (33.5) | 444 (33.6) | 342 (33.3) | 350 (33.5) | 321 (34.4) | 419 (33.9) |
| **CDI treatment** |  |  |  |  |  |  |  |  |
| Metronidazole (PO or IV) | 1119 (81.1) | 1078 (81.9) | 1094 (81.9) | 1085 (82.1) | 841 (82.1) | 851 (81.7) | 755 (80.9) | 1009 (81.7) |
| Vancomycin | 110 (8.0) | 105 (8.0) | 107 (8.0) | 104 (7.9) | 79 (7.7) | 79 (7.6) | 77 (8.3) | 97 (7.8) |
| Metronidazole and vancomycin | 90 (6.5) | 88 (6.7) | 88 (6.6) | 86 (6.5) | 68 (6.6) | 72 (6.9) | 65 (7.0) | 82 (6.6) |
| None | 61 (4.4) | 46 (3.5) | 47 (3.5) | 46 (3.5) | 37 (3.6) | 39 (3.8) | 36 (3.9) | 47 (3.8) |
| **CDI outcomes** |  |  |  |  |  |  |  |  |
| ICU admission | 106 (7.9) | 105 (8.0) | 105 (7.8) | 104 (7.9) | 78 (7.6) | 79 (7.6) | 78 (8.4) | 98 (8.0) |
| ICU admission for CDI complications | 39 (2.9) | 39 (3.0) | 39 (2.9) | 39 (3.0) | 26 (2.5) | 25 (2.4) | 31 (3.3) | 36 (2.9) |
| Toxic megacolon | 14 (1.0) | 14 (1.1) | 14 (1.1) | 13 (1.0) | 8 (0.8) | 9 (0.9) | 11 (1.2) | 13 (1.0) |
| Colonic perforation | 2 (0.2) | 2 (0.2) | 2 (0.2) | 2 (0.2) | 2 (0.2) | 2 (0.2) | 1 (0.1) | 2 (0.2) |
| Colectomy/hemicolectomy | 18 (1.3) | 18 (1.4) | 18 (1.4) | 18 (1.3) | 17 (1.7) | 17 (1.6) | 13 (1.4) | 18 (1.5) |
| 30-day all-cause mortality | 169 (12.2) | 157 (12.0) | 167 (12.5) | 155 (11.7) | 113 (11.0) | 115 (11.1) | 113 (12.1) | 151 (12.2) |
| CDI associated 30-day mortality | 54 (4.0) | 54 (4.1) | 54 (4.1) | 54 (4.1) | 31 (3.0) | 32 (3.1) | 35 (3.8) | 48 (3.9) |
| cCDI^b^ | 108 (7.9) | 103 (7.8) | 101 (7.6) | 99 (7.5) | 67 (6.5) | 67 (6.4) | 71 (7.6) | 92 (7.5) |

NOTE: PPIs, proton pump inhibitors; H2-RA, Histamine type-2 receptor antagonists; EIA, enzyme immunoassay; GDH, glutamate dehydrogenase; HCFA, health care facility-associated; PO, per os; IV, intravenous; IQR, interquartile range.

^a^ Each patient could have received more than one class of antimicrobials within 2 months of enrollment.

^b^ In the original cohort, cCDI was defined as one or more of: admission to an intensive care unit for complications associated with CDI, colonic perforation, toxic megacolon, colectomy or hemicolectomy, or CDI was the cause or contributed to death within 30 days after enrollment.
