# Supplementary material for: External validation of clinical prediction rules for complications and mortality following Clostridioides difficile infection
Source: PLoS One. 2019 Dec 17;14(12):e0226672. doi: 10.1371/journal.pone.0226672 (PMC6917260; doi:10.1371/journal.pone.0226672)
Supplement: S2 Table — (DOCX) [file pone.0226672.s004.docx]

**External Validation of Clinical Prediction Rules for Complications and Mortality Following *Clostridioides difficile* Infection**

Beauregard-Paultre et al. 2019

**S2 Table- Frequencies of predictors in the derivation cohort and in data used for the external validation**

| **Study** | **Sample size** | | **Predictors** | **Frequencies n (%)/ Mean±SD/ Median (IQR)** | |
| --- | --- | --- | --- | --- | --- |
|  | **Derivation cohort** | **Validation cohort^a^** |  | **Derivation cohort** | **Validation cohort** |
| **Prediction of complications** | | | | | |
| Na et al. [24] | 263 | 1318 | Age ≥ 65 years | Mean age 66.5±17.4 | 846 (64.19) |
|  |  |  | WBC ≥ 20 x 10^3^ cells/μL | Mean peak 15.5±11.4 | 147 (11.15) |
|  |  |  | Creatinine ≥ 2 mg/dL | Mean peak 1.8±1.8 | 164 (12.44) |
| Hensgens et al. [25] | 395 | 1338 | Age (years) |  |  |
|  |  |  | 50-84 | 275 (70) | 928 (69.36) |
|  |  |  | ≥ 85 | 35 (9) | 214 (15.99) |
|  |  |  | CDI diagnosed in ICU | 19 (5) | 150 (11.21) |
|  |  |  | Recent abdominal surgery | 110 (28) | 163 (12.18) |
|  |  |  | Hypotension | 117 (30) | 57 (4.26) |
|  |  |  | Diarrhoea as reason for admission | 104 (27) | 234 (17.49) |
| van der Wilden et al. [26] | 746 | 1321 | Age >70 years | 350 (46.9) | 694 (52.54) |
|  |  |  | WBC ≥20 or ≤2 x10^9^/L | 191 (25.6) | 206 (15.59) |
|  |  |  | Cardiorespiratory failure | 30 (4.0) | 42 (3.18) |
|  |  |  | Diffuse abdominal tenderness | 127 (17.0) | 588 (44.51) |
| Shivashankar et al. [29] | 487 | 1026 | Age, 10 years increase | Median 62.5 (0.1-103.7) | Median 70.2 (56.7-79.8) |
|  |  |  | WBC ≥ 15x 10^9^/L | NR | 234 (22.80) |
|  |  |  | Narcotic use |  | 90 (8.77) |
|  |  |  | H2-RA or PPI use |  | 662 (64.52) |
|  |  |  | Creatinine ratio > 1.5 |  | 110 (10.72) |
| **Prediction of mortality** | | | | | |
| Kassam et al. [28] | 374,747 | 1045 | Age (years) |  |  |
|  |  |  | 41-60 | 15 762 (20.3) | 238 (22.78) |
|  |  |  | 61-80 | 33 200 (42.7) | 481 (46.03) |
|  |  |  | 81-100 | 23 319 (30) | 232 (22.20) |
|  |  |  | Critical care/ICU admission | NR | 85 (8.13) |
|  |  |  | Acute renal failure |  | 28 (2.68) |
|  |  |  | Diabetes |  | 213 (20.38) |
|  |  |  | Serious comorbidities |  |  |
|  |  |  | Cardiopulmonary disease |  | 515 (49.28) |
|  |  |  | Liver disease |  | 60 (5.74) |
|  |  |  | IBD |  | 57 (5.45) |
|  |  |  | Malignancy |  | 308 (29.47) |
| Butt et al. [27] | 213 | 933 | Serum albumin ≤ 24.5 g/L | Reported in means and medians according to survival status | 240 (25.72) |
|  |  |  | CRP > 228 mg/L |  | 46 (4.93) |
|  |  |  | WCC > 12 x 10^9^/L and respiratory rate > 17/min |  | 292 (31.29) |
| Archbald-Pannone et al. [30] | 362 | 1235 | Charlson’s score | Median 5 (3-7) | Median 3 (1-5) |
|  |  |  | 0-3 points |  | 667 (54.01) |
|  |  |  | 4-6 points |  | 353 (28.58) |
|  |  |  | ≥ 7 points |  | 215 (17.41) |
|  |  |  | WBC (x 10^9^/L) | 11 (6.8-16.6) | Median 10.1 (6.9-14.5) |
|  |  |  | BUN (mmol/L) | 21 (11-35) | Median 6.0 (3.6-11.0) |
|  |  |  | CDI diagnosed in ICU | NR | 149 (12.06) |
|  |  |  | Delirium | NR | 177 (14.33) |

BUN, blood urea nitrogen. CRP, C-reactive protein. H2-RA, histamine type-2 receptor antagonists. IBD, Inflammatory bowel disease. ICU, intensive care unit. NR, not reported. PPI, proton pumps inhibitor. WBC, white blood cells count.

^a^ The sample used for external validation varied according to complete data for predictors of each CPR.
