# Supplementary material for: External validation of clinical prediction rules for complications and mortality following Clostridioides difficile infection
Source: PLoS One. 2019 Dec 17;14(12):e0226672. doi: 10.1371/journal.pone.0226672 (PMC6917260; doi:10.1371/journal.pone.0226672)
Supplement: S3 Table — (DOCX) [file pone.0226672.s005.docx]

**S3 Table- Reported performance of scores (95%CI) and models for prediction of CDI complications**

| **Study** | **Subset**  **(n; % outcome)** | **Cut-off (n patients; %)** | **Observed outcome/score n (%)** | **Sensitivity (%)** | **Specificity (%)** | **PPV (%)** | **NPV (%)** | **Positive**  **LR** | **Negative LR** | **Accuracy (%)** | **AUC** | **OR for**  **1 point increase in score** |
| --- | --- | --- | --- | --- | --- | --- | --- | --- | --- | --- | --- | --- |
| **Na et al. [24]** | **Derivation**  **n = 251; 12.35%** | **0-1 pt** (180;71.71) | 19 (10.55) |  |  |  |  |  |  |  |  |  |
|  |  | **2-3 pts**  (71; 28.29) | 31 (43.66) | 62.0  (48.15-74.14) | 80.09  (74.04-85.03) | 43.66  (32.74-55.23) | 89.44  (84.10-93.13) | 3.12  (2.19-4.43) | 0.47  (0.33-0.68) | 76.49  (70.87-81.31) | NR | NR |
| **Hensgens et al. [25]** | **Derivation**  **n = 395; 11.9%** | **< 0 pt**  (63; 15.95) | 0 |  |  |  |  |  |  |  | 0.78  (0.71-0.85) | NR |
|  |  | **0-1 pt**  (156; 39.49) | 7^a^ (5) | 100 | 18 | 15 | 100 |  |  | 28 |  |  |
|  |  | **2-3 pts**  (121; 30.63) | 20^a^ (17) | 84 | 61 | 24 | 96 | NR | NR | 64 |  |  |
|  |  | **≥ 4 pts**  (55; 13.92) | 21^a^ (39) | 43 | 90 | 39 | 92 |  |  | 84 |  |  |
| **van der Wilden et al. [26]** | **Derivation**  **n = 746; 6.43%** | **< 6 pts**  (618; 82.84) | 1 (0.16) |  |  |  |  |  |  |  | 0.98  (0.96-1.0) |  |
|  |  | **≥ 6 pts**  (128; 17.16) | 47 (36.72) | 97.9 | 88.4 | 36.7 | 99.8 | NR | NR | NR |  |  |
| **Shivashankar et al. [29]** | **Derivation**  **n = 1446; 33.68%** | **-1.6 (NR)** | - | 94 | 22 |  |  |  |  |  |  |  |
|  |  | **-1.4 (NR)** | - | 90 | 31 |  |  |  |  |  |  |  |
|  |  | **-1.2 (NR)** | - | 85 | 36 |  |  |  |  |  |  |  |
|  |  | **-1.1 (NR)** | Probability 25% | 80 | 46 | NR | NR | NR | NR | NR | 0.706 | NR |
|  |  | **-0.8 (NR)** | - | 70 | 61 |  |  |  |  |  |  |  |
|  |  | **-0.4 (NR)** | - | 53 | 76 |  |  |  |  |  |  |  |

AUC, area under the ROC curve. LR, likelihood ratio. NR, not reported. OR, odds ratio estimated with a univariate logistic regression.

^a^ Number of patients who experienced the outcome was calculated using reported percentages.

^b^ A pilot external validation was conducted in a cohort of CDI during a posterior time-frame (2009-2011 vs 2006-2009 for derivation) in one of the participating centers.
