# Supplementary material for: External validation of clinical prediction rules for complications and mortality following Clostridioides difficile infection
Source: PLoS One. 2019 Dec 17;14(12):e0226672. doi: 10.1371/journal.pone.0226672 (PMC6917260; doi:10.1371/journal.pone.0226672)
Supplement: S4 Table — (DOCX) [file pone.0226672.s006.docx]

**S4 Table- Reported performance (95%CI) of scores and models for prediction of mortality**

| **Study** | **Subset**  **(n; % outcome)** | **Cut-off**  **(n patients; %)** | **Observed outcome/score**  **n (%)** | **Sensitivity (%)** | **Specificity (%)** | **PPV**  **(%)** | **NPV (%)** | **Positive**  **LR** | **Negative LR** | **Accuracy %** | **AUC** | **OR for**  **1 point increase in score** |
| --- | --- | --- | --- | --- | --- | --- | --- | --- | --- | --- | --- | --- |
| **Kassam et al. [28]** | **Derivation**  **n = 77,776 hospitalizations; 7.93%** | **0 pt** (NR) | NR (1.15) |  |  |  |  |  |  |  | 0.77 | NR |
|  |  | **5 pts** (NR) | NR (4.4) |  |  |  |  |  |  |  |  |  |
|  |  | **10 pts** (NR) | NR (20.8) | NR | NR | NR | NR | NR | NR | NR |  |  |
|  |  | **15 pts** (NR) | NR (48.1) |  |  |  |  |  |  |  |  |  |
|  |  | **18 pts** (NR) | NR (100) |  |  |  |  |  |  |  |  |  |
| **Butt et al. [27]** | **Derivation**  **n = 244; 20.49%** | **0 pt** (169; 79.34) | 16 (9.47) |  |  |  |  |  |  |  | 0.754^b^  (0.67-0.84) | NR |
|  |  | **1 pt** (57; 26.76) | 21 (36.84) | NR | NR | NR | NR | NR | NR | NR |  |  |
|  |  | **2 pts** (15; 7.04) | 10 (66.67) |  |  |  |  |  |  |  |  |  |
|  |  | **3 pts** (3; 1.41) | 3 (100.00) |  |  |  |  |  |  |  |  |  |
| **Archbald-Pannone et al. [30]** | **Derivation**  **n = 362; 16.85%**  Range 9.6-73.2 pts  IQR: 21.4-45.2 | **< 10 pts** (1; 0.28)^a^ | 0^a^ |  |  |  |  |  |  |  | 0.804 (NR)  Optimism =  -0.034 (bootstrap) | 11% increase in odds of death |
|  |  | **10-20 pts**  (22; 6.08)^a^ | 0^a^ |  |  |  |  |  |  |  |  |  |
|  |  | **20-30 pts**  (83; 22.93)^a^ | 1^a^ (1.20) |  |  |  |  |  |  |  |  |  |
|  |  | **30-40 pts**  (103; 28.45)^a^ | 12^a^ (11.65) | NR | NR | NR | NR | NR | NR | NR |  |  |
|  |  | **40-50 pts**  (94; 25.97)^a^ | 24^a^ (25.53) |  |  |  |  |  |  |  |  |  |
|  |  | **50-60 pts**  (38; 10.50)^a^ | 15^a^ (39.47) |  |  |  |  |  |  |  |  |  |
|  |  | **60-70 pts**  (13; 3.59)^a^ | 7^a^ (53.85) |  |  |  |  |  |  |  |  |  |
|  |  | **≥ 70 pts**  (1; 0.28)^a^ | 0 |  |  |  |  |  |  |  |  |  |

AUC/ROC, area under the ROC curve. LR, likelihood ratio. NR, not reported. OR, odds ratio estimated with a univariate logistic regression.

^a^ Frequencies were provided by the authors in personal communication.
